# Progress in 1,3-propanediol biosynthesis

**DOI:** 10.3389/fbioe.2024.1507680

**Published:** 2024-11-29

**Authors:** Boran Li, Wenyan Gao, Yuanyuan Pan, Yongpeng Yao, Gang Liu

**Affiliations:** ^1^ State Key Laboratory of Mycology, Institute of Microbiology, Chinese Academy of Sciences, Beijing, China; ^2^ College of Life Sciences, University of Chinese Academy of Sciences, Beijing, China

**Keywords:** 1,3-propanediol, biosynthetic pathway, microorganisms, synthetic biology, glycerol dehydratase, propanediol oxidoreductase

## Abstract

1,3-Propanediol (1,3-PDO) is one of the important organic chemical materials and is widely used in polyester synthesis, and it also shows great potential in medicine, cosmetics, resins, and biodegradable plastics. So far, 1,3-PDO mainly comes from chemical synthesis. However, the by-products and the side effects during chemical synthesis of 1,3-PDO bring about serious damage to the environment. In recent years, the biosynthetic pathway of 1,3-PDO has been elucidated in microorganisms. Under the action of glycerol dehydratase (GDHt) and propanediol oxidoreductase (PDOR), glycerol can be catalyzed to form 1,3-PDO through the reduction pathway. Compared to the chemical synthesis, the biosynthesis of 1,3-PDO is environmentally friendly but would face the problem of low production. To improve the yield, the native 1,3-PDO producing strains have been modified by genetic engineering, and the biosynthetic pathway has been reconstructed in the model microorganism, *Escherichia coli*. In this review, we summarize the research progress of the 1,3-PDO biosynthesis in microorganisms, and hopefully, it will provide reference for the renewable production of 1,3-PDO in industry.

## 1 Introduction

### 1.1 Polyester and 1,3-propanediol (1,3-PDO)

Polyester is known for its decent physical, chemical, and mechanical functions, and it is a kind of highly tensional fiber which is made to fit the growing need of a substitution for traditional cottons and linens. With the development of industrial production, polyester has a wide application throughout the domestic economy. The usage of polyester fiber could be traced back to 1941, when British scientists found and identified the very first polyester fiber, terylene (Kadirvel et al., 2024). Yet, normal polyester and regular fibers are far away from the industrial requirement due to the rapidly increasing need of clean chemistry as well as the uprising manufactural development. Due to the revealing of novel substitute fiber which is becoming keener to the humanity, scientists have tried multiple ways to create or derive an acceptable alternative with better intensity and duration.

**FIGURE 1:**
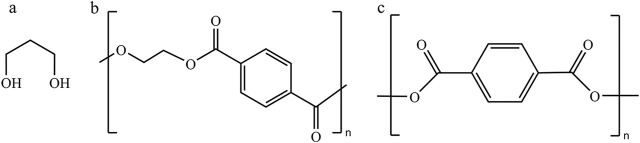
Structures of 1,3-PDO and the polymers derived from 1,3-PDO. **(A)** 1,3-Propanediol (1,3-PDO), **(B)** polypropylene terephthalate (PTT), and **(C)** polyethylene terephthalate (PET).

Terylene is a kind of polyester fiber, which most commonly refers to the high polymer-polyethylene terephthalate (PET) produced by esterification or transesterification of refined pure terephthalic acid (PTA) or dimethyl terephthalate (DMT) and ethylene glycerol (MEG), and commercially known as Dacron (Ahmad et al., 2021). By replacing MEG with 1,3-propanediol (1,3-PDO) for condensation, a new high-performance polyester fiber-polypropylene terephthalate (PTT) can be prepared (Azzouz et al., 2003). PTT fiber holds a unique molecular structure with high resilience. Simultaneously, PTT fiber also has excellent resistance capacity to pollution and wrinkle, and many other characteristics (Rath et al., 2023), which has broad application prospects. 1,3-PDO alongside the refined terephthalic acid, the crucial material for the preparation of PTT fiber, has attracted a great deal of attention from both scientific institutes and industrial manufacturers. Among these two, preparation and manufacture of the refined terephthalic acid are mature, which is already one of the largest binary carboxylic acids with over 50 million tons of domestic output in 2021 alone (Noor et al., 2016). On the other hand, the production of 1,3-PDO is relatively immature, with less than 1 million tons of domestic output, where the price is still too high to fit the industrial needs. Given the current status of unsatisfying production of 1,3-PDO, producing this chemical in high yield becomes the first priority (Figure 1).

## 2 The synthetic approaches of 1,3-PDO

1,3-PDO is not only an important organic chemical material for polyester synthesis but it also shows great potential in medicine, cosmetics, resins, and biodegradable plastics. In the past few decades, various strategies have been applied to produce 1,3-PDO. 1,3-PDO is the simplest member of propane-1,3-diols, consisting of propane where one hydrogen from each methyl group is substituted by a hydroxy group (Zhu et al., 2022). Due to its important applications, 1,3-PDO synthesis has attracted great attention. As a chemical compound with a versatile bulk nature, 1,3-PDO can easily be synthesized via either chemical or biological approaches. Moreover, with the increasing need and application of polyester fiber PTT, the potential value of 1,3-PDO would be much higher in future.

### 2.1 Chemical approaches of 1,3-PDO synthesis

**FIGURE 2:**
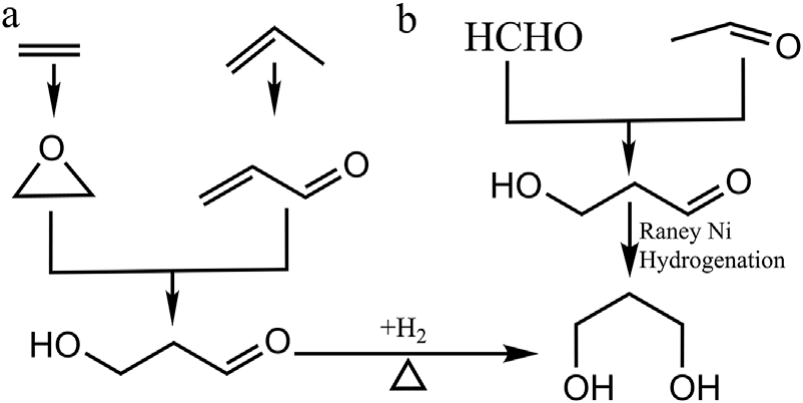
Chemical approaches of 1,3-PDO synthesis. **(A)** In the C_2_ component from the Shell process, ethylene is converted to 3-hydroxylpropanal (3-HPA) during a chemical process called hydroformylation, followed by the catalytic hydrogenation of 3-HPA to finally form 1,3-PDO. In the Degussa process, it initiates from hydration a unique C_3_ component, acrolein: a chemical obtained by the oxidation of propylene, to form 3-HPA which is further hydrogenated to 1,3-PDO. **(B)** the reactants including formaldehyde (HCHO) and acetaldehyde (CH_3_CHO) can be converted to 3-HPA directly via a liquid phase self-aldol condensation reaction.

The chemical approaches of 1,3-PDO synthesis mostly originated from two main routes (Figure 2): one is the C_2_ component from the Shell process where ethylene is converted to 3-hydroxylpropanal (3-HPA) during a chemical process called hydroformylation, followed by the catalytic hydrogenation of 3-HPA to finally form 1,3-PDO. The other route is the Degussa process which initiates from hydration of a unique C_3_ component acrolein, a chemical obtained by the oxidation of propylene, to form 3-HPA which is further hydrogenated to 1,3-PDO (Kurian, 2005). Recently, some other methods forming 1,3-PDO via chemical approaches have been identified, where the reactants including formaldehyde (HCHO) and acetaldehyde (CH_3_CHO) can be converted to 3-HPA directly via a liquid phase self-aldol condensation reaction that holds a crucial role in organic synthesis by the formation of novel carbon–carbon bonds (Jiang et al., 2022).

Other than using simple aldehydes like formaldehyde or acetaldehyde as the initial reactant, glycerol as the main by-product of biodiesel has a promising prospect of being the initial reactant of the 1,3-PDO synthesis. Therefore, one method has been applied using the catalytic glycerol hydrogenolysis reaction (da Silva Ruy et al., 2021), initiated by the dehydration from glycerol to 3-HPA on its acid sites, then followed by a simultaneous addition of hydrogen atom on the metallic sites (Yang et al., 2018). Moreover, there are novel strategies for the catalytic system of the glycerol hydrogenolysis process. Until now, the most effective route in the 1,3-PDO production in the aqueous phase is the use of heterogeneous catalysts originated by noble metals (Fan et al., 2017). For instance, using metal catalysts (Pt/WO_x_/Al_2_O_3_) for the selective hydrogenolysis of glycerol toward 1,3-PDO has been proven in 2015 (García-Fernández et al., 2015). The glycerol regio-selective hydrogenolysis of 1,3-PDO is further tested to be one of the most desirable routes. Some other studies have applied a siliceous mesocellular foam supported platinum catalyst to catalyze the hydrogenolysis due to the high surface area as well as the large pore size (Cheng et al., 2021).

Although chemical approaches have significantly improved the industrial production of 1,3-PDO, the total amount of targeting product is far from meeting the increasing need of both manufacturer and industrial cooperatives. In addition, the by-products and the side effects bring about serious damage to the environment; thus, it is an urgent need to develop environmentally favorable approaches for 1,3-PDO production.

### 2.2 Biological approaches of 1,3-PDO synthesis

It has been found that many microorganisms possess the ability of conversion of glycerol to 1,3-PDO (Hao et al., 2008). Combined with synthetic biology, genome editing, and metabolic modification, the biological approaches have shown significant advantage in 1,3-PDO production, such as high yield and less by-products. Moreover, the increasing application of biodiesel provides abundant crude glycerol as the main by-product. In summary, the promising prospect of biological approaches for 1,3-PDO synthesis is sticking out a mile.

#### 2.2.1 Molecular mechanism of 1,3-PDO biosynthesis

**FIGURE 3:**
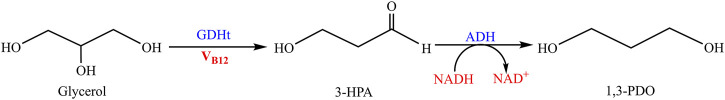
Biosynthetic pathway of 1,3-PDO. Glycerol is converted to 3-hydroxypropanaldehyde (3-HPA) under the catalysis of glycerol dehydratase (GDHt). Then, 3-HPA is catalyzed by alcohol dehydrogenase (ADH) and aldehyde dehydrogenase (ALDH). The former will consume NADH to produce NAD^+^ and the latter will consume NAD^+^ to produce NADH. The ending products are 1,3-PDO and 3-hydroxypropanate (3-HP), respectively.

Like chemical approaches, the biosynthesis of 1,3-PDO is also originated from glycerol, which goes through two possible reactions, including oxidation and reduction in microorganisms. In oxidation reaction, glycerol is converted into various intermediates like dihydroxyacetone (DHA), dihydroxyacetone phosphate (DHAP), phosphoenolpyruvate (PEP), pyruvate, and acetyl-CoA, and finally enters the TCA cycle. Through the TCA cycle, oxidation of glycerol provides sufficient energy and power for the microbial growth. However, some of the by-products from pyruvate and acetyl-CoA such as acetate, lactate, butyrate, and 2,3-butanediol (2,3-BDO) had been identified during the past research, which are all depended on the microorganisms targeted for 1,3-PDO production, and the decrease of by-products was carried out by the control of reactants, enzymes, and genetic engineering. On the other hand, reduction is the predominant scheme of 1,3-PDO biosynthesis (Figure 3), where the glycerol is preliminarily converted to 3-HPA under the catalysis of an enzyme called glycerol dehydratase (GDHt) (Daniel et al., 1998). 3-HPA is further converted to the final product, 1,3-PDO, via an enzyme called propanediol oxidoreductase (PDOR) (Zheng et al., 2006). 3-HPA, the intermediate of 1,3-PDO biosynthesis as mentioned above, has a great application in food preservation and pharmaceutical industry due to its cellular toxicity (Park et al., 2017), and it is important to restrain the toxicity from harming the microorganisms, so generally, the GDHt is the rate-limiting enzyme throughout the total reaction scheme of glycerol reduction (Daniel et al., 1998).

As the key enzyme in the reduction of glycerol, GDHt varies in the structure and function through different microorganisms. Some GDHts require vitamin B_12_ as the crucial cofactor during the reaction, notably the Gram-negative bacteria *Klebsiella pneumoniae*, a microorganism that can produce 1,3-PDO from glycerol as well as some species of *Clostridium* sp. (Macis et al., 1998), and some different cofactors other than vitamin B_12_ are needed for GDHt activity within different microorganisms, like S-adenosyl methionine (SAM) being a specific cofactor of GDHt in *Clostridium* sp. (Walls et al., 2022). In general, GDHt has very short duration, which means it will lose its catalytic activity accompanied by the progress of reaction because of the irreversible breakage process, being accompanied by the conversion of enzyme bound adenosylcobalamin to an alkyl or thiol-cobalamin. Hence, it needs a glycerol dehydratase reactivator to regain its activity (Ushio et al., 1982). The genes encoding reactivator are normally called glycerol dehydratase reactivator (Gdr), which react with GDHt by exchanging the damaged adenosyl moiety for intact vitamin B_12_ through their catalytic activities. In its molecular structure, the reactivator is similar to the well-known GroEL and Hsp70 protein, both of which are the members of molecular chaperone (Liao et al., 2003).

**FIGURE 4:**
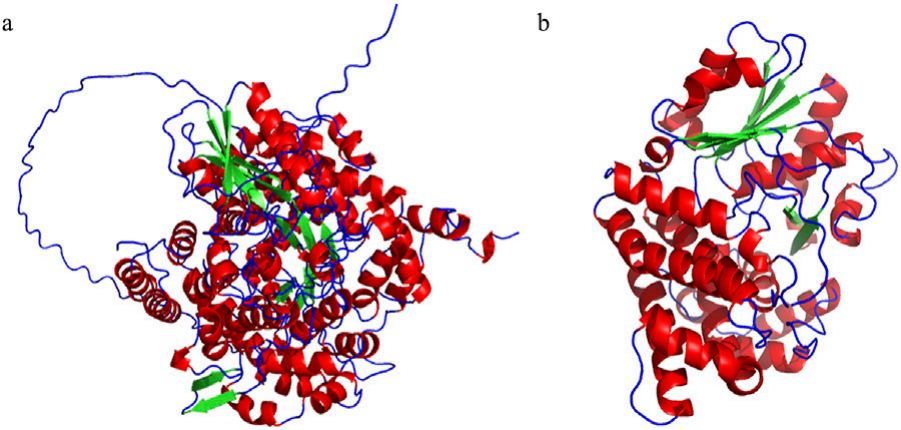
Molecule structures of GDHt and PDOR. **(A)** Structure of DhaB123, which belongs to the family of GDHt, found in *Klebsiella pneumoniae*. There are three subunits originated from three different ORFs, respectively. **(B)** Structure of YqhD, which belongs to the family of PDOR, catalyzing the reaction from 3-HPA to 1,3-PDO; this enzyme is a dimer of two subunits with relatively low specificity yet high reaction activity compared to the other PDORs.

The other key enzyme, PDOR, catalyzes the conversion of 3-HPA to 1,3-PDO and requires ferric ions (Fe^2+^) as a cofactor, and also consumes the reducing forces like NADH or NADPH in catalysis (Zheng et al., 2006). Based on the previous studies, the enzyme specificity of PDOR is relatively low, from which some kind of alcohol oxidoreductase could also catalyze the reaction; that is because of the isofunctional capacity from propanediol oxidoreductase, with the difference in the cofactors as well as the type of reducing forces. It has been reported that not just diol oxidoreductase some other enzymes like aldehyde reductase and alcohol reductase also catalyze this reaction, which are designated as the hypothetical oxidoreductase (HOR) (Figure 4).

#### 2.2.2 Microorganisms for the conversion of glycerol to 1,3-PDO

Some microorganisms utilize glycerol as the sole or main carbon source in order to gain energy for their growth. It has been nearly 90 years since people found that glycerol can be fermented from some anaerobic bacteria. *Clostridium pasteurianum* is the first reported microorganism which can use glycerol to attain other products. During the past decades, certain microorganisms including *Klebsiella*, *Citrobacter*, *Enterobacter*, *Bacillus*, *Lactobacillus*, *Pseudomonas*, and *Clostridium* have been identified of being able to produce diols like 1,3-PDO and 2,3-BDO (Ainala et al., 2013; Barbirato et al., 1996; Guo et al., 2017; Leja et al., 2014; Maervoet et al., 2012; Narcizo et al., 2023).

Among those species with the capability of producing 1,3-PDO, *K. pneumoniae* is considered to have the most advantage due to its clear genetic background as well as the rapid growth with glycerol as the carbon source under both aerobic and anaerobic conditions. Moreover, *K. pneumoniae* is capable of producing vitamin B_12_ (Ashok et al., 2013), which is an important coenzyme of GDHt, which is helpful during the process of synthesizing the precursor of 1,3-PDO. In *K. pneumoniae*, like most microorganisms mentioned above, glycerol is metabolized *via* two pathways including the oxidative pathway and/or the reductive pathway. In the reductive pathway, enzyme GDHt from *K. pneumoniae* has three subunits, namely, DhaB1, DhaB2, and DhaB3 (Xu et al., 2009). GDHt throughout various species of *Klebsiella* genera is reported to have high homology. As mentioned above, vitamin B_12_ plays an important role in the reaction of GDHt, in which the enzyme will eventually end in the mechanism-based inactivation. In *K. pneumoniae*, the reactivators of GDHt are referred to GdrAB; in specific, the genes encoding GdrAB are called *dhaG* (*orfZ*) and *dhaF* (*orfX*), which will reactivate GDHt under the presence of ATP and Mg^2+^/Mn^2+^ ions (Lee et al., 2018). Additionally, the by-products while producing 1,3-PDO in *K. pneumoniae* vary, such as 2,3-BDO, succinate and CO_2_, affecting the final yield of 1,3-PDO.

*Clostridium bifermentans*, *C. pasteurianum*, *Clostridium beijerinckii*, and *Clostridium butyricum* can produce 1,3-PDO from glycerol under anaerobic fermentation (Altafini et al., 2022; Wischral et al., 2016), with an advantage of not requiring aggressive agitation (Guo et al., 2017). In these species, glycerol undergoes an oxidative route under the dehydration of NAD^+^-dependent GDHt called DhaD, resulting in dihydroxyacetone, which will be further phosphorylated by dihydroxyacetone kinase (DhaK), and enters the central metabolism like glycolysis and TCA cycle. Meanwhile, glycerol is converted to 3-HPA through the reduction catalyzed by the vitamin B_12_-dependent GDHt (DhaB, DhaC, and DhaE) in some species, and 3-HPA is further reduced to the final product under the catalysis of an NADH-dependent PDOR (DhaT) (Biebl and Spröer, 2002). The four key enzymes in the reductive pathway are encoded by the *dha* regulon, where the total expression is induced by the presence of glycerol itself or dihydroxyacetone. Additionally, *C. butyricum* and *C. pasteurianum* hold the most similar conversion modules in 1,3-PDO biosynthesis. In specific, *C. butyricum* is believed to hold more promising production prospects, and it is reported that additional polysaccharides like xylose or arabinose would enhance the titer of 1,3-PDO production in *C. butyricum* (Apiwatanapiwat et al., 2018). On the other hand, *C. bifermentans* is able to produce 1,3-PDO under microaerobic conditions (Leja et al., 2014) with less by-product. However, the relatively lower biomass and longer seed cultivation time limited the industrial application of *Clostridium* sp.

*Citrobacter* sp. (Maervoet et al., 2012) is also identified as a native 1,3-PDO-producing strain despite its relatively lower yield than *K. pneumoniae* (Hao et al., 2008), but it shows a higher conversion rate due to the inner elimination of by-products such as 2,3-BDO and ethanol. *Citrobacter freundii* is deemed to be the predominant strain for 1,3-PDO production (Boenigk et al., 1993). *C. freundii* and *K. pneumoniae* are preliminarily classified as opportunistic pathogens that would cause infection on hosts under certain conditions. *C. freundii* is less pathogenic than *K. pneumoniae*, which gives it an advantage on the further metabolic modification. Just like *C. butyricum*, the relatively lower yield is still withholding their potentials.

*Lactobacilli* sp. is another microorganism that can produce 1,3-PDO naturally. Considering safety, some species of the *Lactobacilli* family have been studied on their potential of producing 1,3-PDO because the *Lactobacilli* family are generally considered as GRAS. However, *Lactobacilli* cannot grow in a culture medium with glycerol as the sole carbon source and cannot produce 1,3-PDO from glucose (Zhu et al., 2022). However, *Lactobacilli* grow well and produce a decent amount of 1,3-PDO when the culturing medium contains glycerol and sugar together. The species like *Lactobacillus reuteri*, *L. diolivorans*, and *L. panis* can produce 1,3-PDO from glycerol with supplement carbon source like glucose; in particular, *L. diolivorans* shows good industrial application potential with a titer of up to more than 85 g/L of 1,3-PDO (Stevens et al., 2013).

*Enterobacter agglomerans*, a microorganism, was first identified and isolated from the anoxic mud of wastewater distillation digester (Barbirato et al., 1995) and has been reported to synthesize 1,3-PDO via the glycerol conversion. However, *Enterobacter agglomerans* produces large amounts of formate, which might decrease the pH value and eventually lead the reaction to the acid production route, lowering the yield of 1,3-PDO. Another example is *Enterobacter aerogenes*, but it is normally studied for its biological hydrogen production ability, with 1,3-PDO serving as a by-product (Pachapur et al., 2015).

In summary, although various microorganisms can produce 1,3-PDO naturally, *K. pneumoniae* and *C. butyricum* are the most promising strains which show good glycerol utilization ability as well as high yield. *K. pneumoniae*, as a facultative anaerobic microorganism, shows the simplicity of its experimental operation. Moreover, the well-established modification tools and methods on *K. pneumoniae* give it more potential of enhancing the productivity of 1,3-PDO.

### 2.3 Improving production of 1,3-PDO in microorganisms

With the increase in global demand, the native strains are no longer able to meet the requirements of industrial production of 1,3-PDO. Mutation breeding can improve the 1,3-PDO production, but there is limitation without revealing its biosynthetic pathway. Fortunately, we have known the biosynthetic pathway of 1,3-PDO and can handle it by using genetic methods and synthetic biology strategies. Genetically modifying the native 1,3-PDO producing strains has significantly improved the yield of 1,3-PDO. Meanwhile, inserting key genes for the 1,3-PDO biosynthetic pathway into the model strain *Escherichia coli*, which originally does not produce 1,3-PDO, has successfully achieved the production of 1,3-PDO.

Whereas *Corynebacterium glutamicum*, a microorganism that meets the criterion of GRAS, has also been applied for the production of 1,3-PDO, and due to the naturally possessed glycerol kinase (encoded by *glpK*), modification of *C. glutamicum* has become one applicable route on the production of 1,3-PDO.

#### 2.3.1 Mutation breeding of the 1,3-PDO producing strains

Mutation breeding is a traditional method to improve the yield of compounds in microbial strains. Basically, mutation breeding includes physical and chemical mutagenesis. Physical methods such as UV, γ-rays, X-rays, and particle radiation are generally used in physical mutagenesis. The chemicals like azides and alkylation agents are used in chemical mutagenesis.

It has been reported that ultraviolet light as the mutagenic agent can improve the yield of 1,3-PDO in *K. pneumoniae*, where the induced culture first went a product acclimatization by 1,3-PDO, then it was treated by ultraviolet light to improve the production. There are also some works using chemical mutagenic agents like ethyl methanesulfonate (EMS) or N-ethyl-N-nitrosourea (ENU) to apply mutagenesis of *C. pasteurianum* (Gallardo et al., 2017). ENU has the advantage of providing multiple and wide range of mutations and eventually causes high frequency mutation. But the cellular toxicity and additional oxidation stress of ENU limit its further application.

In general, physical mutagenesis methods have lower costs and easier operation, but the mutation rate is relatively low. Chemical methods are mostly using mutagenic agents that are poisonous and carcinogenic. Ergo, it is crucial to develop novel clean and recyclable mutagenesis methods.

A novel physical mutagenesis method called atmospheric and room temperature plasma (ARTP) has been used in mutagenesis (Li et al., 2024), which has a better performance than UV radiation and chemical methods. In fact, mutagenesis including atmospheric plasma, which creates an instant plasmatic environment by breaking down the gas between the electrodes at atmospheric pressure, has gradually been developing due to the non-toxicity and operation simplicity. Moreover, the combination of ARTP with chemical mutagenic agents will surely apply more efficient mutants. Yang et al. reported that using ARTP along with N-methyl-N′-nitroN-nitrosoguanidine (NTG) to treat *C. butyricum* XYB11 achieved a desirable mutant with much higher 1,3-PDO tolerance and production (Yang et al., 2019).

Despite the various merits and developments of induced mutagenesis, its limitation is obvious, such as the inefficiency of screening methods and long preparation time of acclimatization and mutation.

#### 2.3.2 Genetically engineered microorganisms for 1,3-PDO production

Genetically modifying microorganisms has been an effective route to improve the production of the target compounds in various fields. *Via* this method, the metabolic pathway of the native strain is refactored toward a direction that is more appropriate of obtaining products by enhancing the expression of genes targeted to the final product synthesis and eliminating the expression of genes for by-products which hinder the total production of the target compound.

In the native producers of 1,3-PDO, modifications mostly focus on the by-product elimination. For instance, in *K. pneumoniae*, the glycerol utilization is a dismutation process which will form various by-products like lactate, acetate, and 2,3-BDO. Therefore, blocking the biosynthesis of 2,3-BDO and so on can concentrate the metabolic flow and further lead to a higher yield of 1,3-PDO. Lee et al. (2018) constructed such a mutated strain (Δ*ldhA*Δ*pflB*Δ*budA*Δ*glpK*Δ*dhaD*Δ*dhaKLM*), where the predominant gene *budA* (encoding 2,3-butanediol dehydrogenase) for 2,3-BDO production was deleted, as well as the genes for lactate and acetate producing. Moreover, the genes attributed in glycerol assimilation (*glpK* and *dhaD*) were deleted and mannitol was introduced as a co-substrate. Finally, the molar yield of 1,3-PDO was improved significantly to 0.76 mol/mol glycerol. Cui et al. (2014) constructed two 2,3-BDO defiant strains (Δ*budA*Δ*budB* and Δ*budA*Δ*budB*Δ*ldhA*) along with the overexpression of *dhaT* encoding PDOR which could utilize the excess NADH and eventually enhance the 1,3-PDO production in *K. pneumoniae*. In another reported work, Oh et al. (2012) identified the effect caused by the deletion of lactate dehydrogenase (*ldhA*), in which they constructed two deletion modules, one was *ldhA* and the other was *budB* encoding the acetolactate synthase ALS which was involved in the production of 2,3-BDO. Results showed that the deletion of *ldhA* eliminated the by-product lactate and therefore enhanced the 1,3-PDO production. The deletion of *budB* caused the redistribution of metabolic flux, where the consumption of NADH was decreased and led to reducing the by-product 2,3-BDO, eventually increasing the yield of the target product, 1,3-PDO (Zhou et al., 2019).

Compared to the native 1,3-PDO producers such as *K. pneumoniae*, *C. butyricum*, and *C. freundii*, the model microorganisms like *E. coli* and *C. glutamicum* have more advantages. For instance, these microorganisms, unlike the native producers, can be cultured under mild conditions, and they rarely cause severe diseases or harm to human and environment. Moreover, these strains have more industrial potentials due to the simplicity of culturing and genetic operations. So far, there are numerous precedents of using *E. coli* to produce valuable chemicals in industry scale.

DuPont company developed a commercial biological route through *E. coli* strain metabolic engineering and fermentation for 1,3-PDO production using glucose originated from corn (Wang et al., 2024). They applied genetic modification, introducing the genes for glycerol and 1,3-PDO biosynthesis, further deleting the genes for the biosynthesis of by-products such as lactate and acetate in *E. coli*. Fermentation was applied by using glucose from corn lysis as the sole carbon source, resulting in 75 kt/a of 1,3-PDO.

Following DuPont’s methods, various bio-based routes have been applied to synthesis 1,3-PDO from corn-originated glucose. A recombinant of *E. coli* with introducing the genes encoding the glycerol-3-phosphate dehydrogenase (*gpd*) and glycerol-3-phosphatse (*gpp*) was constructed. Genes *gpd* and *gpp* originally identified from *Shewanella cerevisiae* and their products carry out the conversion of glucose to glycerol (Nakamura and Whited, 2003). However, there was too much energy consumed during this carbon source conversion and the by-products were synthesized at a higher level, reducing the yield of 1,3-PDO. To solve this problem, Frazão et al. (2019) established an alternative route for 1,3-PDO synthesis from glucose by using the synthetic pathway, leading to the production of the non-natural metabolite (L)-2,4-dihydroxybutyrate (L-DHB) that departs from the TCA cycle intermediate malate through three non-natural enzymatic reaction steps. In this route, a pathway from glucose to malate then to DHB and eventually leading toward 1,3-PDO was constructed. In general, they have constructed two sets of recombinant strains based on *E. coli* K12-MG1655, in which strain A contains the malate-DHB pathway and strain B contains the 1,3-PDO production pathway. As a result, they have achieved a 40-fold increased 1,3-PDO titer *via* co-cultivation of these two strains. However, despite the low cost of using corn-based glucose, the use of food still raises some concerns, especially on the aspect of sustainability. On the other hand, glycerol, as a residue from biofuel as well as a renewable organic carbon source, would be a more reliable source of producing 1,3-PDO sustainably.

In a previous study, Tong et al. (1991) constructed a modified *E. coli* strain carrying the key 1,3-PDO biosynthetic enzymes from *K. pneumoniae*, and put their focus on the crucial *dha* regulon. The 1,3-PDO biosynthetic pathway in *K. pneumoniae* was under the regulation of *dha* regulon, which was induced by dihydroxyacetone without the presence of exogenous electron acceptors like oxygen, fumarate, or nitrate (Forage and Foster, 1982). But the enzymes under *dha* regulon are not directly involved in the biosynthesis of 1,3-PDO, the physiological reason is most likely that the crucial cofactor NAD^+^ was regenerated by the DHA branch of the *dha* regulon. Under normal conditions, *E. coli* cannot grow on mere glycerol anaerobically without any exogenous electron acceptor as mentioned above (Sprenger et al., 1989). By using the cosmid containing genes under the *dha* regulon from *K. pneumoniae*, including *gdrAB*-*dhaB123*, *dhaT*, and the gene encoding DHA kinase, a recombinant *E. coli* strain with the ability of producing 1,3-PDO was constructed.

Due to the heterogeneity of the genes from other species, the 1,3-PDO yield is relatively low. In 2003, an NADPH-dependent aldehyde reductase YqhD was identified from *E. coli* and it was used for producing industrial preferable diols such as 1,3-PDO or 1,2-PDO. YqhD was immediately used for 1,3-PDO production by various research workers. Qi *et al.* constructed a strain based on *E. coli* BL21 that contained the GDHt and its reactivator from *K. pneumoniae*, and overexpressed the *yqhD* from *E. coli* BL21 and got a 8.0 g/L yield of 1,3-PDO (Zhang et al., 2006). Wong *et al.* developed more indel modules in *E. coli* NSK001 (Wong and Jantama, 2022). For instance, the GDHt operon from *K. pneumoniae* was rearranged and the native *E. coli* NADPH-dependent aldehyde reductase gene *yqhD* was simultaneously overexpressed as well. The genes encoding lactate dehydrogenase (LdhA), acetate kinase (AckA), and pyruvate formate lyase (PflB) were deleted, whereas the *cat-sacB* cassette (containing the chloramphenicol acetyltransferase-levan sucrose gene) from *Klebsiella oxytoca* KMS005 and the kanamycin resistant gene were amplified by PCR. Result showed that this engineered strain *E. coli* NSK015 (∆*ldhA*::*gdrAB*-*dhaB123*∆*ackA*::FRT ∆*pflB*::*yqhD*∆*frdABCD*::*cat-sacB*) was capable of producing 1,3-PDO at 36.8 g/L with astonishing 0.99 mol/mol glycerol, which is the highest conversion rate reported.

As mentioned above, some of the 1,3-PDO synthetic routes did not seem to have a decent yield. Hence, it is needed to explore other possible routes, such as the co-substrate and the co-production. The co-substrate not only uses glycerol as carbon source but also uses other chemicals as carbon source such as glucose, xylose, and starch. In this way, the modified microorganisms have more rapid growth, and it provides a more suitable condition for 1,3-PDO production. In the co-production route, more than one microorganism is mixing in a certain ratio, which would maximize the utilization of the limited carbon source.

**FIGURE 5:**
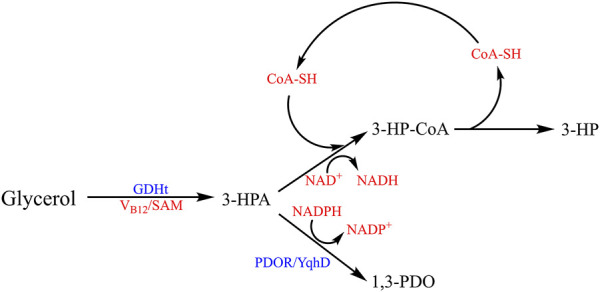
Metabolic pathways of co-producing 1,3-PDO and 3-HP. The crucial intermediate 3-HPA is used to produce 3-hydroxypropionic acid (3-HP) under the catalyzation of GabD4 via oxidation reaction, and the NAD^+^ is reduced to NADH which could be converted to NADPH. Meanwhile, 3-HPA is catalyzed to form 1,3-PDO by PDOR/YqhD in the presence of NADPH.

For example, Tang *et al.* constructed an *E. coli* strain carrying the gene encoding GDHt and its reactivating factor (DhaB1 and DhaB2) from *C. butyricum*, *yqhD* encoding PDOR from *E. coli*. They used a temperature-sensitive plasmid pBY220 as the transformation vector and obtained the *E. coli* K-12 recombinant (*dhaB1*::*dhaB2*::*yqhD*). In the production phase, two-stage fermentation was applied, where the first stage with glucose as the carbon source accumulated the cell pellet and the second stage initiated the glycerol utilization. Throughout the two-stage fermentation process, the original glucose-based media would undergo an apparatus triggered phase perfusion, in which the media would be gradually replaced by the glycerol-based media, and 1,3-PDO was synthesized at the second stage. This result showed a yield of 104.4 g/L 1,3-PDO with more than 0.74 mol/mol conversion ratio (90.2% g/g) (Tang et al., 2009a). Qi *et al.* put their attention on the co-production of 1,3-PDO and some other valuable industrial products and synthesized them simultaneously (Figure 5). They have chosen another crucial by-product from the glycerol dismutation reaction, 3-hydroxypropionic acid (3-HP). 3-HP, like 1,3-PDO, is a simple chemical with a good potential industrial value, which has been evaluated to be about $10 billion by 2022 (Zhang et al., 2021). The crucial intermediate 3-HPA, the product of the first step of microbial glycerol reduction, was basically utilized to produce 3-HP. To achieve this goal, a succinate-semi-aldehyde dehydrogenase (GabD4) from *C. necator* was used for the conversion of 3-HPA into 3-HP *via* oxidation reaction. In this process, NAD^+^ would be reduced to NADH, regenerating NADH, which could be converted to NADPH under enzymatic catalysis. For efficient conversion, the gene encoding soluble transhydrogenase (SthA) was knocked out, whereas the gene encoding pyridine nucleotide transhydrogenase (PntAB) was over-expressed, altering the reaction flows toward the formation of NADPH, which is crucial for glycerol reduction. With this strategy, a genetically modified strain (Δ*sthA*::UTR*glpK*::*yqhD*-*gabD4*-*pntAB*::*dhaB123*-*gdrAB*) was constructed based on *E. coli* W3110. With glycerol and corn starch liquor as the carbon source, it finally yielded 140.50 g/L of total 3-HP and 1,3-PDO in 0.85 mol/mol conversion rate (Zhang et al., 2023).

However, *E. coli* is technically not a GRAS microorganism and it might cause mild diarrhea or even severe gastrointestinal illness due to its naturally occurring endotoxins. Therefore, other microorganisms were used to replace *E. coli*. As reported recently, an efficient *C. glutamicum* strain has been designed for producing 1,3-PDO from glucose, where two different glycerol modules were constructed. In detail, they have introduced *gpd* and *gpp* from *S. cerevisiae* to convert DHAP to DHA *via* a dephosphorylation reaction (Nakamura and Whited, 2003), then further to form glycerol. Another inner set of genes *hdp*A and *gla*A was used for the conversion of DHAP to glycerol-3-phosphate, then toward glycerol. As the glycerol accumulation was sufficient, they introduced the 1,3-PDO biosynthetic module, initiated by propanediol utilization dehydratase (encoded by *pdu*) and its reactivator combined with PDOR encoded by *yqhD*, and finally achieved the high titer of 1,3-PDO. Meanwhile, the genes for the biosynthesis of by-products were knocked out. These genes, including *ldhA* for lactate, *poxB* for acetate, *pyk* for pyruvate excessive accumulation, *ppc* for the oxaloacetate originated from phosphoenolpyruvate, *ald* for 3-HP synthesis, and *adhA* for the reverse process of 1,3-PDO to 3-HPA, were deleted or inhibited. As a result, a yield with 110.4 g/L of 1,3-PDO by 0.42 g/g glucose as the conversion ratio has been obtained (Li et al., 2022).

### 2.4 Co-culture of microorganisms for 1,3-PDO production

As mentioned above, it has been reported that the engineered strains were used for synthesizing 1,3-PDO, but the metabolic stress undoubtedly will affect the bacterial growth during fermentation. To release the stress, a co-culture system containing two different strains which carry the partial metabolic pathway separately was designed and used (Table 1).

**TABLE 1:** Strategies of producing 1,3-PDO *via* co-culture.

Host strain	Carbon source	Fermentation type	Concentration (g/L)	Yield (g/g)	Productivity (g/L*h)	Reference
*Klebsiella* sp. strain YT7 and *Shewanella oneidensis* MR1	Glycerol	Fed-batch	62.9	0.53	1.07	Hartlep et al. (2002)
*K. pneumonia* and *C. butyricum*	Glycerol	Fed-batch	52.08	0.49	1.80	Sun et al. (2022)
*K. pneumonia* and *E. coli*	Glycerol	Fed-batch	43.20	0.39	0.98	
*K. pneumoniae*, *E. coli*, and *C. butyricum*	Glycerol	Fed-batch	81.39	0.51	N/A	
*Candida krusei* and *K. pneumoniae*	Glycerol and glucose	Fed-batch	21.2	0.19	0.30	Zeng et al. (1994)
*C. acetobutylicum* and *S. cerevisiae*	Glycerol	Fed-batch	25.5	0.24	N/A	Mendes et al. (2011)
*E. coli* Rosetta-*dhaB12* and *E. coli* BL21	Glycerol and glucose	Fed-batch	41.6	0.67	0.69	Yun et al. (2021)

The co-culture system has successfully converted glucose to 1,3-PDO, in which it includes a leading strain that converts the initial carbon source (normally glucose) to glycerol, and a second strain that can naturally utilize glycerol to produce the targeting products. Hartlep et al. (2002) used the recombinant *E. coli* as the leading strain and *K. pneumoniae* as the second strain for 1,3-PDO production. The result showed a good synthetic rate at 2.0 g/L/h; however, the total yield of 1,3-PDO was still low.

To further improve the conversion rate as well as the product yield, *Klebsiellla* sp. strain YT7 was isolated and produced 11.30 g/L of 1,3-PDO with glycerol as the carbon substrate. Furthermore, *Shewanella oneidensis* MR-1 was added and a co-fermentation culture was reached, which can produce 1,3-PDO to the maximum of 62.9 g/L (Wang et al., 2023). Sun *et al.* mixed the cultures from the most predominant 1,3-PDO-producing strains *Klebsiella pneumonia* and/or the most widely used modified strain *E. coli* together with *C. butyricum*, and the highest production of 1,3-PDO reached 81.39 g/L under strict anaerobic fermentation, whereas even at non-strict anaerobic condition, there still was 77.68 g/L of 1,3-PDO (Sun et al., 2022). In addition, yeast *Candida krusei* was used to produce glycerol from glucose in the co-culture system, and one of the native 1,3-PDO producers, *K. pneumoniae*, has been widely used for 1,3-PDO biosynthesis from glycerol. The growth conditions of *C. krusei* and *K. pneumoniae* were identified separately and the mixture was tested. The result showed that the growth of *K. pneumoniae* was decreased with the addition of glucose. However, as the constant utilization of glucose by *C. krusei*, the inhibition of glucose toward *K. pneumoniae* will be reduced. Eventually, the 1,3-PDO production reached to 21.2 g/L, nearly as twice as much of solely cultivating *K. pneumoniae*. In another study, *S. cerevisiae* HC42 was used due to its tolerance to 200 g/L of glucose. Combined with *S. cerevisiae* HC42 for producing glycerol, engineered *C. acetobutylicum* was used as the main producer of 1,3-PDO, and the result showed 25.5 g/L of 1,3-PDO by a 0.24 g/g yield (Mendes et al., 2011).

### 2.5 Bioprocess control

Other than genetically engineering, optimization of bioprocess holds a great deal of importance as it would further improve the final yield. Tang *et al.* on the other hand achieved high production *via* a process called two-stage fermentation. During the first stage, the fermentation has been set as aero-bioculture, where glucose was used as the sole carbon source. The main objective is to increase the cell amount. At the second stage, a discontinuous procedure was applied for replacing the spent culture medium with the anaerobic fermentation medium. Then, the anaerobic medium including trace salt and glycerol is pumped with nitrogen as the protective gas for creating the anaerobic environment. In this method, the final yield of 1,3-PDO has been enhanced to 104.4 g/L with a productivity of 2.61 g L^-1^ h^-1^, and the total fermentation time scale was only 40 h, which is shorter than that of any of the reports (Tang et al., 2009b).

On the other hand, Wong *et al.* applied a process called the two-pulsed continuous feeding strategy (2CF). In this strategy, the feeding periods were at 24–56 and 80–112 h incubation time, with the glycerol feeding rate, glucose feeding rate, and feeding ratio of glycerol to glucose at 1.04 g h^-1^, 1.08 g h^-1^, and 1:1.04, respectively (Wong et al., 2023). With this bioprocess control strategy as well as the former modification, they have successfully enhanced the production of 1,3-PDO to 60 g/L, with a productivity of 0.42 g L^-1^ h^-1^. Moreover, this process could reduce the cost and hence better contribute to the large-scale production of 1,3-PDO.

Most recently, Wang *et al.* have developed the two-stage continuous fermentation by kinetics modification, where a novel three-stage continuous fermentation was designed aiming for 1,3-PDO production in *C. butyricum*. The first stage provided seeds with good robustness for the following two stages, and the second stage mainly aimed to facilitate the production of 1,3-PDO. For the third stage, the concentration and yield of 1,3-PDO was further enhanced while reducing the residual glycerol as much as possible. By applying this method, a yield of 80.05 g/L of 1,3-PDO with the productivity of 3.67 g L^-1^ h^-1^ has been achieved, and the introduction of kinetic modeling is of great importance to the optimization of multi-stage continuous fermentation (Wang et al., 2024).

## 3 Discussion and future prospects

### 3.1 Reinforcement of the reducing force for 1,3-PDO production

**FIGURE 6:**
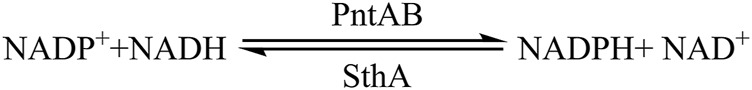
Biotransformation route between NADH and NADPH. The endogenous reducing force would undergo a self-conversion between NADH and NADPH via two key enzymes (PntAB and SthA) catalyzing a reversible reaction. For efficient conversion, the gene encoding soluble transhydrogenase (SthA) was knocked out, whereas the gene encoding pyridine nucleotide transhydrogenase (PntAB) was over-expressed.

The reductive process of glycerol is the key route of synthesizing 1,3-PDO, whereas it needs reducing force like NADH (reductive coenzyme Ⅰ) and NADPH (reductive coenzyme Ⅱ) (Figure 6). As reported, introduction or overexpression of *yqhD* is generally used. Unlike the native PDOR which normally requires NADH, YqhD needs NADPH as the electron acceptor. Thus, increasing the amount of NADPH endogenously is practically needed. Introduction of the genes for generating or preserving NADPH is a reasonable route to reinforcement of NADPH. Introducing the NADPH-dependent dehydrogenases was first considered. For example, formate dehydrogenases (FDHs) have been applied in various conditions. However, the utilization of FDHs is limited by their undesired cofactor preference, which targets NAD^+^ more than NADP^+^ in biosynthesis (Alpdagtas and Binay, 2021). Yet as reported, the attempts to switch or alter the cofactor preference have not met a desirable result.

Some enzymatic systems can increase the NADPH production, and the membrane bound pyridine nucleotide transhydrogenase encoding by *pntAB* in *E. coli* catalyzes the conversion between NADH and NADPH. However, overexpression of *pntAB* cannot increase the accumulation of NADPH due to its reversible catalytic ability. SthA or referred as UdhA, which is a soluble transhydrogenase catalyzing the opposite reaction of PntAB, mainly converts NADPH to NADH (Sauer et al., 2004). To further enhance the accumulation of NADPH, Chen *et al.* applied a *sthA* knockout strain of *E. coli* which immobilized part of the conversion from NADPH to NADH and furthermore overexpressed *pntAB* by increasing its copy number. The engineered strain gave a decent titer of NADPH and 1,3-PDO. In this method, only a part of NADH conversion was inhibited; the natural routes such as glycolysis and TCA cycle were all intact. The novel glycerol reduction pathway has the capability of consuming and regenerating NADPH. Through introducing the novel glycerol reduction pathway, the final yield and concentration of 1,3-PDO have significantly increased (Li et al., 2022).

Another potential enzyme that has been identified successfully is PpnK, a polyphosphate/ATP-NAD^+^ kinase originated from *Mycobacterium tuberculosis* (Mori et al., 2005). PpnK can form NADP^+^ *via* the phosphorylation and be regarded as a key enzyme in the NADP^+^ synthesis as well as in protection against oxidative stress throughout various anabolic and/or biosynthetic pathways. By introducing or overexpressing *ppnK*, the titer of targeting products was increased, and overexpression of *ppnK* also caused the accumulation of *p*-coumaric acid by nearly 2-fold (Qiu et al., 2024). Similarly, Hong et al. (2016) have used another NAD^+^ kinase Pos5 originated from *S. cerevisiae* to enhance the final product, poly-3-hydrobutyrate, from 2.30 g/L to 3.82 g/L.

In conclusion, the cofactor NADPH is involved in various anabolic reactions and its activity has been considered to be a crucial role. Introducing NADPH-dependent formate dehydrogenase or some other enzyme systems, such as PntAB, UdhA, and PpnK from *E. coli*, and Pos5 from *S. cerevisiae*, will either convert NADH to NADPH through phosphorylation or it might ultimately result in enhancing the 1,3-PDO production.

### 3.2 Transport of glycerol and excretion of 1,3-PDO

In the native 1,3-PDO producers like *K. pneumoniae*, *C. butyricum*, and *C. freundii*, glycerol is assimilated generally by passive diffusion, and then utilized by the corresponding enzymatic systems. However, in the modified strains like *E. coli*, the situation would be different. In the engineered *E. coli*, the utilization of glycerol throughout the reaction route is enhanced by the glycerol uptake facilitator, GlpF (Voegele et al., 1993), and then it flows into the glycerol utilization pathway. But the efflux of final product 1,3-PDO, which is a crucial step of the product enhancement, has not been fully reported. The inner accumulation of 1,3-PDO might eventually hinder its further synthesis. Moreover, it is believed that the identification and introduction of a certain efflux system will further increase the total yield of 1,3-PDO in the engineered strains.

Some previous works indicated that the *mex* system containing a 12-transmembrane-segment protein located in the cytoplasmic membrane of *Pseudomonas aeruginosa* would be responsible for the efflux of diol (Gotoh et al., 1999). The *mex* system or Mex pump is one of the best studied resistance-nodulation-cell division (RND) pumps, which could be subdivided into three clusters phylogenetically. The first one contains the heavy metal efflux protein, the second participates in secretion of nodulation factors, and the third represents the multidrug efflux pumps including the Mex pump. Substrate specificity of the Mex pump is determined by the cytoplasmic membrane component (MexB, MexD, MexF, and MexY) of the tripartite efflux pump system (Guan et al., 1999).

The *mex* systems differ in their substrate and expression preferences, and the IMP members MexB and MexF originated from the multidrug efflux system of *P. aeruginosa* show contrary cellular growth effect, which indicates the complexity and the broad substrate specificity of these efflux pumps.

Notably, as reported, MexF dramatically enhanced GDHt activity and promoted 1,3-PDO titer and glycerol conversion rate up to 74.0 g/L and 0.62 mol/mol in the recombinant *K. pneumoniae*, respectively (Guan et al., 1999). It has already been reported that the overexpression of *glpF* improved 1,3-PDO titer in *K. pneumoniae*. However, co-expression of the endogenous *glpF* and *mexF* did not further improve 1,3-PDO production (Teng et al., 2022). The results provided novel information about the applications of the uptake of glycerol and the efflux of product, which might lead the further secretion of 1,3-PDO and therefore improve the final yield of 1,3-PDO.

### 3.3 Enhancing 1,3-PDO production by replenishing of TCA cycle under anaerobic or microaerobic environments

TCA cycle is a crucial metabolic pathway that needs to be accomplished at the aerobic condition. Oxygen does not participate in the cycle; however, it plays an important role in the consecutive oxidation of reducing coenzymes such as NADH or NADPH located in the electron transport chain. During biosynthesis of 1,3-PDO, the reducing force is a crucial factor as mentioned above, but most microorganisms with natural or modified ability of producing 1,3-PDO mostly belong to anaerobic or facultative anaerobic microorganisms in which the intensity of TCA cycle would undergo a significant decrease. With that scenario, replenishing TCA cycle under anaerobic or microaerobic condition might be a novel pathway of producing related products (Vo and Park, 2022).

The electron regulation occurs in anaerobic or microaerobic conditions compared to aerobic ones. As reported, the Fnr system showed its unique function in anaerobic regulation on multiple terminal oxidoreductase operons (Gunsalus, 1992). The Fnr system is normally silent in aerobic cultures in phenotypical aspect. However, the *fnr* gene seems to be a negative regulator of many TCA cycle genes in response to anaerobiosis. Deletion of the *fnr* gene would cause a 2.5-fold elevation in the activity of isocitrate dehydrogenase (encoded by *icd*-*lac*Z), thus enhancing the TCA cycle in anaerobic conditions (Chao et al., 1997).

The Arc system, on the other hand, is mainly responsible for the regulation of numerous gene expressions which are dependent on the availability of oxygen and other electron acceptors in the culture environment. Fnr regulates the gene expressions under anaerobic condition, whereas the Arc system regulates the expression of numerous operons under both aerobic and anaerobic conditions, as well as microaerobic condition. The Arc system, composed of ArcA which is the cytosolic response regulator and ArcB which is the membrane bound sensor kinase, regulates the TCA cycle depending on the oxygen level or redox state. The TCA cycle is further activated in the *arcA* gene knockout mutant as compared with the *arcB* mutant (Shalel-Levanon et al., 2005). As the TCA cycle is the source of energy for the cell, its activation together with upregulation of other gene expression related to the TCA cycle is attractive for the improvement of the cell growth and useful metabolite production. The activation of the TCA cycle together with limited respiratory capacity caused NADH accumulation, which may eventually enhance the production of the TCA cycle-related biosynthesis.

### 3.4 Optimization of bioprocess

A novel possible route might be the bioconversion of resting cell, which is the bioprocess different from the traditional batch fermentation or fed-batch fermentation, notably on the transformation target. Bioconversion (or biotransformation) deals with microbial enzymatic transformation of the substrate into the product with a limited number of one or a few enzymatic reactions, whereas fermentation includes several reactions, which are often complex in nature (Lin and Tao, 2017). As in for 1,3-PDO, there is no report applying bioconversion on this chemical specifically; however, 3-hydroxypropionaldehyde (3-HPA), one key intermediate of 1,3-PDO production, has been reported of using bioconversion for its production (Chen and Chen, 2013). Moreover, one high value product, limonene-1,2-diol, has also been reported that it could be converted from limonene by bioconversion (Sales et al., 2019). Therefore, using bioconversion as a potential route of producing 1,3-PDO should have a great deal of prospective.

## 4 Conclusion

To replace the current chemical route of 1,3-PDO production from petroleum in the industry, intense effort has been put to develop the biological route. Modification of the native 1,3-PDO-producing strains has significantly improved the yield through enhancing the 1,3-PDO biosynthetic pathway and blocking the by-product biosynthesis. However, strict cultivation conditions and inconvenient in genetic manipulation of the native strains hinder the rational modification of these strains and their application in the industry. Based on the synthetic biology strategy, reconstruction of the 1,3-PDO biosynthesis pathway in the model microorganisms has shown promising prospects. With a deeper understanding of the 1,3-PDO biosynthesis process and a wider exploration of the components such as glycerol dehydratase (GDHt) and propanediol oxidoreductase (PDOR), we believe that renewable manufacturing based on 1,3-PDO biological synthesis routes will inevitably replace the chemical synthesis routes which use petroleum as raw materials.
